# Sleep and chronotype influence aerobic performance in young soccer players

**DOI:** 10.3389/fphys.2023.1190956

**Published:** 2023-05-11

**Authors:** Andrea Ciorciari, Antonino Mulè, Lucia Castelli, Letizia Galasso, Fabio Esposito, Eliana Roveda, Angela Montaruli

**Affiliations:** ^1^ Department of Biomedical Sciences for Health, University of Milan, Milan, Italy; ^2^ I.R.C.C.S. Ospedale Galeazzi—Sant’Ambrogio, Milan, Italy

**Keywords:** sleep, chronotype, soccer, aerobic performance, football, endurance, sport

## Abstract

People can be classified into three chronotypes (CT): morning-type (M-type), Neither-type (N-type) and Evening-type (E-type). M-types perform better in the morning, E-types in the evening. It seems that bad sleep worsens physical performance. The impact of sleep and CT on specific sports and populations is unclear. Therefore, we wanted to assess agility, strength and endurance in young soccer players in relation to their sleep and chronotype. 58 players (13–19 years) were recruited. Sleep and CT were assessed by questionnaires. The physical trial was performed at 8:30 a.m. and 6:00 p.m., and included three tests to determine agility, strength and endurance. The sample was classified by CT as *M-types* (n = 11), *N-types* (n = 29) and *E-types* (n = 18). Furthermore, they were categorized as *people with Good Sleep/Wake quality* (GSW, n = 28) and *people with Bad Sleep/Wake quality* (BSW, n = 30). Comparing the three CTs in the aerobic test, M-types performed better in the morning (*p* = 0.01), while E-types in the evening (*p* < 0.001). GSW performed better than BSW (*p* = 0.019) in the aerobic test in the p.m. session. These results underline the difference in aerobic power between M-and E-types during the morning and evening session; moreover, they show a difference in p.m. aerobic performance according to sleep quality.

## 1 Introduction

Physical performance is determined by integrating several physiological and psychological variables (Raglin, 2001; Joyner and Coyle, 2008). Many of these factors are subordinate to rhythmic fluctuations, due to the circadian clocks that regulate the biology of organisms (Winget et al., 1985; Bonaconsa et al., 2014; Montaruli et al., 2017; Duglan and Lamia, 2019). It follows that athletes' ability can be affected by the moment of the day/month/year when the performance is required (Ayala et al., 2021).

The phenotypic expression of circadian rhythms, resulting from genetic, social and environmental factors, can be defined as Circadian Typology (CT) or chronotype (Horne and Ostberg, 1976; Adan et al., 2012; Montaruli et al., 2021). People can be classified into three major typologies: Morning-type (M-type), Neither-type (N-type) and Evening-type (E-type). M-types are more active in the initial stages of the day, wake up and go to bed early and are usually more synchronized with our morning society. E-types’ peculiarities are the opposite of M-types; therefore, they show deregulated rhythms and are typically bad sleepers. Lastly, N-types show mixed characteristics, straddling the other two chronotypes (Partonen, 2015). CT has been shown to be strictly dependent on age (Roenneberg et al., 2007): in particular, children are typically M-types, but during the adolescence they tend to switch their daily routine later, reaching their peak of lateness at the age of 20. Subsequently, a slow and lifelong transition to earlier preferences occurs.

Although all three chronotypes are subject to daily fluctuations in terms of physical performance, it seems that the most influenced are the M- and, especially, the E-types. When exercise is performed in synchrony with their biological clock, the former tends to perform better in the earlier stages of the day, while the latter in the later parts (Vitale and Weydahl, 2017; Facer-Childs et al., 2018). Consequently, practicing physical activity out of synchrony (i.e., an E-type at morning) results less performing and more fatiguing: M-types tend to have a higher effort perception in the evening, while E-types in the morning (Mulè et al., 2020; Malek et al., 2023). Researchers have been trying to find out the moment of the day in which, regardless of CT, the maximum performance occurs, and it appears to be in the early evening, at the peak of core body temperature (Teo et al., 2011). It is well-known that body temperature undergoes fluctuations over 24 h, reaching its peak in the late afternoon (Refinetti, 2010). It has been found that this can generate an increase in the metabolism, by boosting the use of carbohydrates instead of fats as an energy source, improving muscle compliance, and facilitating the formation of acto-myosin bridges in the sarcomere. All these factors lead to a better performance (Mansingh and Handschin, 2022).

As previously anticipated, CT is closely related to sleep. Sleep is a physiological state characterized by poor sensory responsiveness and reduced movement. It is a key process for human homeostasis as it is able to regulate molecular mechanisms related to the cognitive, psychological and metabolic spheres (Hobson, 2005). The relationship between sleep and physical activity on health is well demonstrated (Memon et al., 2021; Mulè et al., 2022). In terms of performance, this relation is more controversial (Watson, 2017), specifically in youth (Riederer, 2020). Nevertheless, it has been observed that bad sleep habits, such as restriction/deprivation or bad sleep quality, tend to worsen physical performance (Dolezal et al., 2017) especially in endurance and prolonged exercises (Fullagar et al., 2015; Castelli et al., 2022). Furthermore, Facer-Childs and Brandstaetter showed that sports performance could depend on the interaction between chronotype and sleep, i.e., the time elapsed since waking up (Facer-Childs and Brandstaetter, 2015). According to this study, peak performance time was approximately 5.5 h and 6 h after awakening for, respectively, M- and N-type athletes, while E-types reached their peak performance approximately 11 h after their awakening.

It is important to clarify that not only physical performance and daily activities can be affected by sleep and CT, but also sleep and CT can be influenced by daily physical activity (Vitale et al., 2019; Whitworth-Turner et al., 2019). Practicing physical activity in the early morning may anticipate the acrophase of Rest-Activity circadian Rhythm (RAR), while exercising in the later parts of the day may lead to a delayed RAR and sleep onset.

Often, success in top level competition is determined by small variations in performance (Brager et al., 2022). For this reason, researchers always look for any potential factor which may lead to an improvement in sport-specific abilities. Training an athlete at a specific time of the day, meeting his daily preferences to reach higher level of performance, may induce an upgraded quality of physical preparation (Hill et al., 1988). Consequently, understanding the importance of complementary factors, such as CT and sleep, on morning and evening performances can be critical in establishing training schedules. Therefore, the aim of this study was to verify whether agility, strength and endurance can be affected by sleep quality and chronotype in young soccer players.

## 2 Materials and methods

### 2.1 Participants

Fifty-eight young male soccer players (age = 15.16 ± 1.73 years; BMI = 20.61 ± 2.24 kg/m^2^) were recruited from a non-professional team located in the suburbs of Milan. All the roles (goalkeeper, defender, midfielder, striker and playmaker) were well represented. Nineteen subjects were 18–19 years old, but all of them were students, and their school routine was similar to the minors’ one. Participants with pathologies that could compromise the physical performance and/or sleep quality were not included. Before the beginning of the study all participants and, if minors, their parents, received an explanation of the purpose, methods, risks, and benefits of the study. They were then asked to sign a written informed consent for the participation. The study was carried out in accordance with the tenets of the 1964 Declaration of Helsinki and approved by the Ethical Review Board of the University of Milan.

### 2.2 Chronotype and sleep assessment

Following a brief anamnesis and the assessment of anthropometric data, the subjects were asked to compile two questionnaires: the *Morningness-Eveningness Questionnaire* (MEQ, Horne and Ostberg, 1976) and the *Mini-Sleep Questionnaire* (MSQ, Zoomer et al., 1985). The MEQ is a validated tool used to assess the chronotype. It has 19 items that aim to investigate the daily habits of the participants; the total score is then used to categorize the subjects into the three circadian typologies previously described: *M-types*, *N-types* and *E-types*. The MSQ is a 10 items survey used to evaluate the seven-day sleep quality prior to compilation. According to their score, the subjects are divided in four categories: people with *good sleep-wake quality* (score: 10–24); people with *mild sleep-wake difficulties* (score: 25–27); people with *moderate sleep-wake difficulties* (score: 28–30); people with *severe sleep-wake difficulties* (score: >30). Considering our sample size, all the subjects with no sleep disturbances were identified as *people with Good Sleep/Wake quality* (GSW, score between 10–24), while those who had mild/moderate/severe sleep problems were identified as *people with Bad Sleep/Wake quality* (BSW, score above 24). Both questionnaires were previously described to the participants who, under the supervision of a researcher, compiled them.

### 2.3 Agility, strength and endurance assessment

The data collection was carried out in the month of November 2022. The participants were asked not to practice vigorous physical activity the 2 days before the test sessions. In order to avoid any bias, they and the investigators were not informed about their questionnaire outcomes. The physical trial was performed in two different moments with at least 1 day of rest in between: in the morning, at 8:30 a.m., and in the evening, at 6:00 p.m., to exacerbate any difference due to the chronotype. Both sessions were identical and included three tests to determine agility, strength and endurance, performed in this order. These parameters were chosen because representative of the physical tasks demanded to soccer players, both during training sessions and matches. The trial lasted about 1 hour and was always preceded by a specific warm-up. The choice of the tests was made according to their reliability, validation and practicality.

The agility was tested by the *Illinois Agility Test* (IAT, Cureton. 1951). The participants were asked to run as fast as possible from the starting line to the end of a course, following a precise path passing through different cones. In every experimental session, the time was always recorded by the same researcher: the shorter it was, the better the test resulted. The Sargent Jump Test (SJT, de Salles et al., 2012) assessed the strength of the lower limbs. After raising one arm next to a wall and marking the point the hand touched, the subjects were asked to maximally jump to touch the same wall at the highest point they could. Then, the difference between the two marked points was determined to evaluate, in centimeters, the height of the jump and the strength of the subject. The aerobic endurance was estimated by the *6 Minutes Run Test* (6MRT, Mänttäri et al., 2018). The participants were asked to run as far as possible at a constant speed for 6 min. The farther they went, the better the results. At the end of both AM and PM sessions, participants were asked to indicate their general fatigue using the *RPE Borg CR10 scale* (Borg, 1982).

### 2.4 Statistical analysis

The participants were categorized in terms of CT (M-type, N-type, E-type) and sleep/wake quality (GSW and BSW). According to the main goal of the study, the comparisons were made between the GSW vs*.* BSW and the three CTs in all the tests’ results (IAT, SJT, 6MRT) in both the conditions (AM and PM). The normality of the distribution of the data was assessed by Shapiro-Wilk Test. The Levene’s Test was used to evaluate the homogeneity of variance of the data. ANOVA and *t*-test were performed to compare the age between the groups. Considering that CTs and GSW/BSW did not differ in age, we decided to not include this variable in the analyses. Two-way ANOVA was performed to investigate the interaction effect of CT and sleep on performance. The comparisons between GSW and BSW were made by independent *t*-test in order to evaluate the effect of sleep on performance of each session. The comparison between AM and PM tests in each group (GSW and BSW) were made by paired *t*-test. A repeated measures ANOVA (AM vs*.* PM) was used to compare the three CTs in every test and in Borg scale. If necessary, post-hoc analysis (Bonferroni adjusted) was performed. The strength of the effect of CT and sleep on each test was quantified by evaluating the effect size according to Cohen (Cohen, 1992). The statistical analyses were performed using SPSS Statistics version 28 (IBM SPSS Statistics for Windows, Armonk, NY, United States of America: IBM Corp), setting the statistical significance to *p* = 0.05.

## 3 Results

**FIGURE 1:**
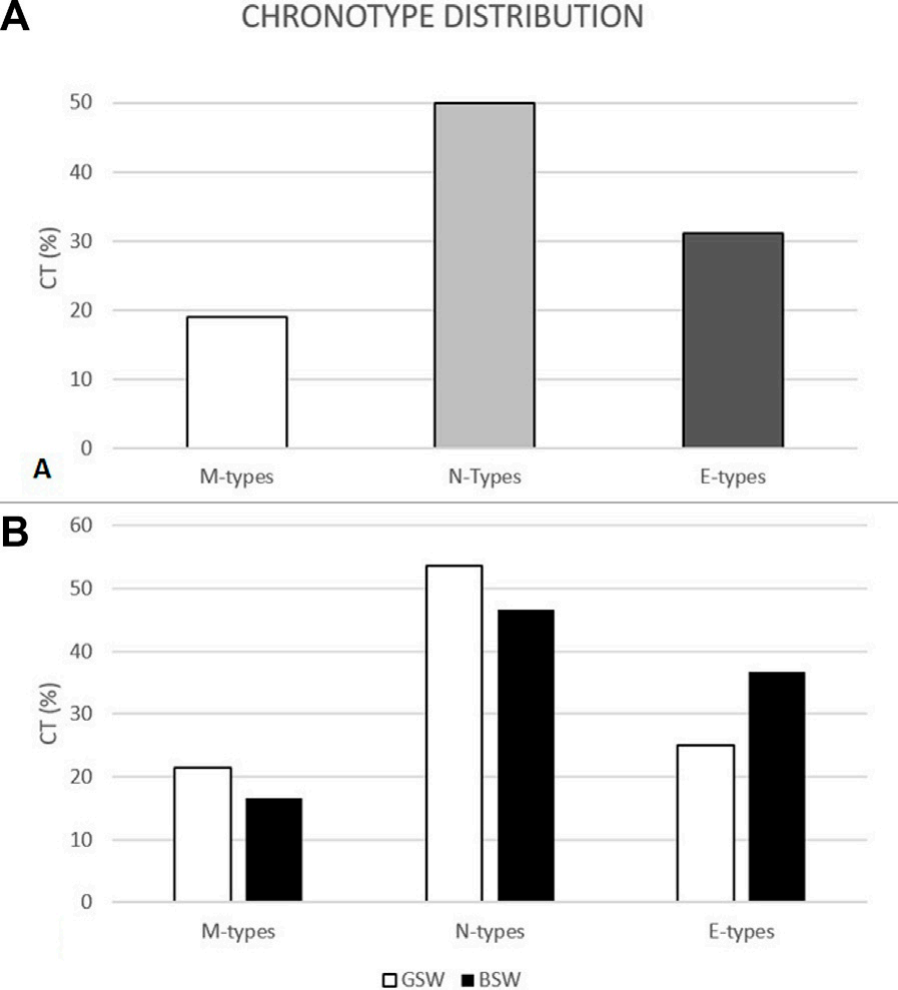
Chronotype distribution of the sample (M-types = 11; N-types = 29; E-types = 18) **(A)**; chronotype distribution (%) within the people with Good Sleep/Wake quality (GSW) and people with Bad Sleep/Wake quality (BSW) categories **(B)** *.*

The total sample (n = 58; age = 15.16 ± 1.73 years; BMI = 20.61 ± 2.24 kg/m^2^) was classified by CT as *M-types* (n = 11; 19%), *N-types* (n = 29; 50%) and *E-types* (n = 18; 31%), as displayed in Figure 1A; Figure 1B shows the distribution of the CTs within the groups of *people with Good Sleep/Wake quality* (n = 28: 21% M-types; 54% N-types; 25% E-types) and *people with Bad Sleep/Wake quality* (n = 30: 16% M-types; 47% N-types; 37% E-types). M-, N- and E-types did not differ in age, as well as GSW and BSW.

**FIGURE 2:**
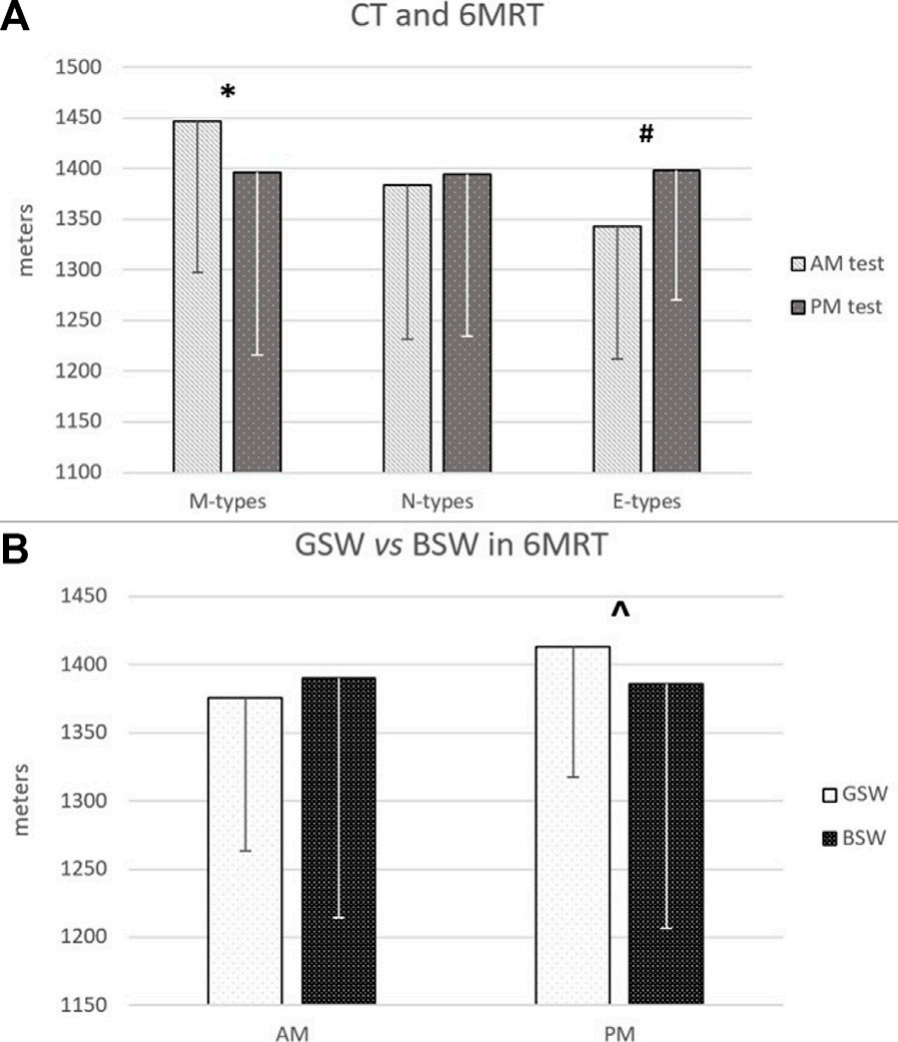
Comparison between M-, N- and E-types in the two 6MRT sessions **(A)**; comparison between people with Good Sleep/Wake quality (GSW) and people with Bad Sleep/Wake quality (BSW) in the two 6MRT sessions **(B)**. * M-types AM vs. M-types PM; # E-types AM vs. E-types PM; ^ GSW PM vs. BSW PM = p < 0.05.

Two-way ANOVA (CT x sleep) analysis showed no statistically significant interaction effect on performance in every test (6MRT, SJT, IAT). Table 1 shows the comparisons between the three CTs in the three tests in both sessions (AM and PM). Repeated Measures ANOVA showed statistically significant effects of CT only in 6MRT (F = 9.737; df = 2; *p* < 0.001; ηp2 = 0.261). M-types performed better in the morning (*p* = 0.01; d = 0.5), while E-types in the evening (*p* < 0.001; d = 0.4) (Figure 2A). N-types performance did not differ in the two sessions. The between-factors comparisons, both in AM and PM sessions, showed no difference. Table 1 also shows the results of the comparisons between CTs in Borg scale score in the two sessions (repeated measures ANOVA, F = 8.197; df = 2; *p* < 0.001; ηp2 = 0.230). E-types had a higher score in the AM session (*p* < 0.001; d > 0.8) compared to the PM session, while M-types had a higher score in the PM compared to the AM session (*p* = 0.05; d = 0.4).

**TABLE 1:** Comparisons between the three CTs in the three tests and BORG score. Mean scores and standard deviations for 6-Minute Run Test (6MRT), Illinois Agility Test (IAT), Sargent Jump Test (SJT) and Borg score, at 8:30 a.m and 6:00 p.m., for M-, N-, and E-types. * M-types AM vs. M-types PM (6MRT); ^#^ E-types AM vs. E-types PM (6MRT); ° M-types AM vs. E-types AM (BORG); ^ M-types AM vs. M-types PM (BORG); ^§^ E-types AM vs. E-types PM (BORG) = *p* < 0.05.

	6MRT (m) AM	6MRT (m) PM	IAT (s) AM	IAT (s) PM	SJT (m) AM	SJT (m) PM	BORG (score) (AM)	BORG (score)(PM)
M-types	1447.00	1396.27	17.37	17.33	2.77	2.76	7.45	8.09
	± 49.70*	± 180.15*	± 0.55	± 0.43	± 0.14	± 0.14	± 1.12^^,°^	± 1.64^
N-types	1383.55	1394.41	17.60	17.36	2.74	2.74	8.34	8.28
	± 152.01	± 160.39	± 0.88	± 0.98	± 0.12	± 0.14	± 1.20	± 1.53
E-types	1343.50	1398.67	17.74	17.38	2.76	2.76	9.17	8.11
	± 31.43^#^	± 127.91^#^	± 0.64	±0.53	± 0.14	± 0.16	± 1.04^§,°^	± 1.23^§^

Furthermore, in the AM session, M-types showed a significantly lower Borg score than E-types’ (*p* < 0.001; d > 0.8), while in the PM session no statistically significant difference was observed between M- and E-types. N-types’ Borg score differed neither in the two sessions nor in comparison with the other groups. The independent *t*-test (Table 2) showed that GSW performed better than BSW (*p* = 0.019; d = 0.2) in the 6MRT in the PM session (Figure 2B); no statistically significant differences emerged in AM session and in the other tests. No difference in sleep quality was observed in the comparison (one-way ANOVA) between the three CTs.

**TABLE 2:** Comparisons between GSW and BSW in the three tests. Mean scores and standard deviations for 6-Minute Run Test (6MRT), Illinois Agility Test (IAT) and Sargent Jump Test (SJT), at 8:30 a.m and 6:00 p.m., for people with Good Sleep/Wake quality (GSW) and people with Bad Sleep/Wake quality (BSW). * GSW PM vs. BSW PM (6MRT) = *p* < 0.05.

	6MRT (m) AM	6MRT (m) PM	IAT (s) AM	IAT (s) PM	SJT (m) AM	SJT (m) PM
GSW	1375.64 ± 112.06	1413.14 ± 95.84*	17.49 ± 0.60	17.23 ± 0.48	2.74 ± 0.15	2.76 ± 0.17
BSW	1390.17 ± 175.76	1385.67 ± 178.89*	17.71 ± 0.88	17.45 ± 0.90	2.76 ± 0.12	2.75 ± 0.13

## 4 Discussion

This study aimed to verify whether chronotype and sleep affect performance in young soccer players, comparing their agility, strength and endurance in morning and evening session. Many studies have explored the influence of sleep on physical performance in general (Winget et al., 1985; Thun et al., 2015; Vitale and Weydahl, 2017), but the results are still unclear; furthermore, young soccer players are little studied in chronobiological terms, and the studies on chronotype on this population are few. Our results showed that aerobic performance, evaluated by the 6MRT, is affected both by sleep and CT. Conversely, no statistically significant differences were shown in strength and agility.

### 4.1 Sleep and physical performance

As regards the sleep, our results showed that GSW performed better than BSW only in the evening test session of 6MRT. These findings fit well with the existing literature on soccer player performance and sleep, as many studies showed a significant effect of sleep on aerobic power, but not on strength and agility. Specifically, in soccer players, Abbott et al. (2022) observed no modification in Counter Movement Jump after a night of sleep restriction. Abedelmalek et al. (2014) showed a reduction of aerobic power in the evening (18:00) after a night of partial sleep deprivation. Pallesen et al. (2017) investigated the effect of sleep restriction on soccer-specific ability, but the results were poor, probably due to the timing of the tests, performed in the early morning.

### 4.2 Chronotype and physical performance

Regarding CT, only in the aerobic test, M-types performed better in the morning and E-types in the evening. These results confirm our previous data (Roveda et al., 2020), underlining the difference in aerobic power between M- and E-types during the morning and evening session. Although our actual data displayed no difference in agility and strength, a clear effect in endurance susceptibility to CT was observed. It is possible that longer efforts may exacerbate better the slight differences due to circadian typologies. Hill et al. (1988) reported a reduction in VȮ_2max_ in E-types collegiate students in a test performed in the morning. Also, Brown et al. (2008) in his study on rowers reported similar results: the aerobic performances were significantly affected while the strength ones were not.

### 4.3 Implications

Our data, regardless of CT, seem to indicate that the subjects identified as BSW are more affected by the poor sleep in the evening aerobic session. This concept could be explained by the results of Facer-Childs et al. (2015), who highlighted how sleep debt affects performance as the hours after the awakening increase. Although CT, sleep and aerobic performance seem closely intercorrelated, it is still difficult to reach a conclusion. Indeed, other studies (Burgoon et al., 1992; Kunorozva et al., 2014) also showed no influence of these parameters in cardiopulmonary tests. Our results allow to better understand the inter-relationship between sleep/CT and performance, and may give practical suggestions to coaches and athletic trainer: nowadays, a lot of young people suffer from bad sleep, and it is possible to think that training athletes with sleep problems in the evening may be less productive. Trainers should be aware of the critical issues that sleep has on performance, and they should inquire about their junior athletes’ sleep schedule. Moreover, it may be useful to exploit these results in the organization of the team in prevision of the games, choosing the most performing athletes according to the time of the matches.

### 4.4 Limitations and future perspectives

The limitations of our study surely include the characteristics of our sample in terms of number, sex (only male) and, in particular, age range. CT is very susceptible to age, especially during the adolescence; a larger sample may have been useful to better distinguish and stratify our subjects. Furthermore, our investigation methods are not as accurate as laboratory tests. Future studies should focus on wider age range and deeper investigation methods, with objective sleep evaluation and physical performance assessed with laboratory tests. Moreover, considering that the role in soccer determines the energy demand of the players, a further categorization of the sample (goalkeeper, defender, midfielder, striker, playmaker) could be performed.

## 5 Conclusion

In conclusion, our results may be useful for planning both the time of training and the choice of players for the matches. Daily fluctuations in performance can be seen only with a magnifying glass. With this in mind, aerobic performance in soccer players can be crucial, and also the small differences exacerbated by sleep and chronotype displayed by this study could separate success from defeat. Inquiring about the sleep schedules of athletes may lead to a better comprehension of their rhythms and help coaches to plan the time of their training session.

## Data Availability

The raw data supporting the conclusion of this article will be made available by the authors, without undue reservation.
